# Preparation of polyclonal anti-*Schistosoma mansoni* cysteine protease antibodies for early diagnosis

**DOI:** 10.1007/s00253-023-12408-4

**Published:** 2023-02-11

**Authors:** Alyaa Farid

**Affiliations:** Biotechnology Department, Faculty of Science, Cairo University, Giza, Egypt

**Keywords:** *S. mansoni*, ELISA, Cysteine protease, Polyclonal antibody

## Abstract

**Abstract:**

In many parts of the tropics, schistosomiasis is a major parasitic disease second only to malaria as a cause of morbidity and mortality. Diagnostic approaches include microscopic sampling of excreta such as the Kato-Katz method, radiography, and serology. Due to their vital role in many stages of the parasitic life cycle, proteases have been under investigation as targets of immunological or chemotherapeutic anti-*Schistosoma* agents. Five major classes of protease have been identified on the basis of the peptide hydrolysis mechanism: serine, cysteine, aspartic, threonine, and metalloproteases. Proteases of all five catalytic classes have been identified from *S. mansoni* through proteomic or genetic analysis. The study aimed to produce polyclonal antibodies (pAbs) against schistosomal cysteine proteases (CP) to be used in the diagnosis of schistosomiasis. This study was conducted on *S. mansoni-*infected patients from highly endemic areas and from outpatients’ clinic and hospitals and other patients infected with other parasites (*Fasciola*, hookworm, hydatid, and trichostrongyloids). In this study, the produced polyclonal antibodies against *S. mansoni* cysteine protease antigens were labeled with horseradish peroxidase (HRP) conjugate and used to detect CP antigens in stool and serum samples of *S. mansoni-*infected patients by sandwich ELISA.

The study involved 200 *S. mansoni-*infected patients (diagnosed by finding characteristic eggs in the collected stool samples), 100 patients infected with other parasites (*Fasciola*, hookworm, hydatid, and trichostrongyloids), and 100 individuals who served as parasite-free healthy negative control. The prepared pAb succeeded in detecting CP antigens in stool and serum samples of *S. mansoni-*infected patients by sandwich ELISA with a sensitivity of 98.5% and 98.0% respectively. A positive correlation was observed between *S. mansoni* egg counts and both stool and serum antigen concentrations. Purified 27.5 kDa CP could be introduced as a suitable candidate antigen for early immunodiagnosis using sandwich ELISA for antigen detection.

**Key points:**

• *Detection of cysteine protease antigens can replace parasitological examination*.

• *Sandwich ELISA has a higher sensitivity than microscopic examination of eggs*.

• *Identification of antigens is important for the goal of obtaining diagnostic tools*.

## Introduction

Schistosomiasis is a parasite illness that dates back to ancient Egypt. Case detection and subsequent community treatment, morbidity evaluation, and control strategy assessment are all critical for schistosomiasis management (Peng et al. 2008). Direct parasitological methods and indirect approaches (detection of antibodies (Abs) or serum circulating antigens (Ags)) are used to diagnose schistosomiasis (Magaisa et al. 2015).

Because of its cheap operating expenses and survivability under unstable laboratory structure situations, microscopic presentation of the parasite’s eggs in feces and urine remains the most popular approach. However, this method has many disadvantages like time-consuming, significant egg count fluctuations, and low infection rates (Ross et al. 2001). Immunodiagnostic methods, on the other hand, offer significantly better sensitivity and simplicity of use (Ross et al. 2002; Maher et al. 2022), and several antibody tests have been created (van Dam et al. 2004).

Proteases were investigated as goals for immunologic or therapeutic anti-schistosomal drugs due to their critical participation in several phases of the parasite life cycle (Brindley and Pearce, 2007). The peptide hydrolysis process distinguishes five primary protease classes: serine, cysteine, aspartic, threonine, and metallo-protease (Lecaille et al. 2002). Only a few known *S. mansoni* proteases have been assigned function and the great majority of them are digestive proteases involved in the digestion of metabolic food or the penetration of host tissues (Delcroix et al. 2006).

Researchers have collected and purified the excretory/secretory (E/S) products, and evaluated their functional capabilities by culturing adult worms in vitro (Chappell and Dresden, 1987). Cysteine proteases (CPs) were a key component of the E/S products. CP was isolated from E/S products in our investigation, and the purified CP antigens were utilized in rabbit vaccination to produce anti-CP immunoglobulin G (IgG) polyclonal antibodies (pAbs). Sandwich ELISA was performed to diagnose *S. mansoni* infection using the produced IgG pAb. The results were compared to those obtained using standard parasitological examination techniques.

## Materials and methods

### Animals

Before the tests, New Zealand male white rabbits weighing around 3 kg and aged around 4 months were tested and found to be free of *S. mansoni* and other parasite infections. Rabbits were immunized with *S. mansoni* protease worm extract for the generation of pAbs, as reported by Tendler et al. (1991). All experimental procedures and animal maintenance were performed according to the international care and use of laboratory animals’ guidelines, and were approved by the Institutional Animal Care and Use Committee, Egypt. The manuscript reporting adhered to the ARRIVE guidelines for the reporting of animal experiments.

### Parasites

Adult *S. mansoni* worms were recovered by perfusion from infected mice (6–8 weeks) after infection with 50 cercariae each. To eliminate the host blood and contaminating bacteria, phosphate-buffered saline (PBS, pH=7.4) was used to wash worms of *S. mansoni* several times.

### *S. mansoni* CP antigen preparation

Mature *S. mansoni* worms were incubated, for 16 h at 37°C, in Roswell Park Memorial Institute (RPMI) 1640 medium (pH 7.3) that contains glucose (2%), 30mM HEPES (N-2-hydroxyethyl piperazine-N-2-ethanesulfonic acid), and 25 mg/ml gentamycin. The medium was collected after incubation and centrifuged at 15,000 g for 30 min to remove the parasite eggs. The supernatant was aliquoted and kept at −20°C as E/S products. Inhibition of the protease activity of the E/S products of adult fluke was examined using 2.7 μm l^−1^ aportinin (Sigma-Aldrich, Burlington, Massachusetts, USA), 1 pM pepstatin (Sigma-Aldrich, Burlington, Massachusetts, USA), and 1 μM l-trans-epoxysccinyl-leucylamid-(4-guanidino)-butane (E64) (Sigma-Aldrich, Burlington, Massachusetts, USA). 7-amino-4-methylcomarin (Z-Phe-Arg-AMC), a flurogenic substrate, was used to test the activity of CP in each fraction, where a spectrometer (LS50) was used to measure the fluorescent AMC group at exciter (370 nm) and analyzer wavelengths (440 nm). The protein content of the prepared Ag was described by the method of Bradford (1976). Ion exchange chromatography (Sheehan and Gerald 1996), gel filtration chromatography on a Sephacryl S-200 HR column, and sodium dodecyl-sulfate polyacrylamide gel electrophoresis (SDS-PAGE) (Thaumaturgo et al. 2002) were all used to purify CP. Indirect ELISA was utilized to test the reactivity of CP Ags (Engvall and Perlman 1971).

### Production of pAbs

#### Immunization of rabbits for production of pAbs

Rabbit anti-*S. mansoni* antibodies were produced through the immunization of two New Zealand white rabbits with the prepared *S. mansoni* CP Ag. Each rabbit was immunized by the following: (1) the intramuscular injection of the priming dose (1 mg *S. mansoni* CP prepared Ags mixed with complete Freund’s adjuvant (Sigma-Aldrich, Burlington, Massachusetts, USA) by the ratio 1:1), (2) two booster doses (0.5 mg of *S. mansoni* CP prepared Ags emulsified in incomplete Freund’s adjuvant). The 1^st^ booster dose was 2 weeks later after the priming dose; and the 2^nd^ booster dose was 1 week after the 1^st^ booster dose. Blood samples were collected from the animals: (1) prior to the immunization process to make sure that the rabbit was clean from any infection with other parasites, (2) at each following injection (boosting) to measure the titer of produced polyclonal antibodies. After 1 month, the animals were scarified for the collection of blood samples. Serum was fractionated and stored at −20°C until used.

#### Purification of rabbit anti-*S. mansoni* IgG pAb

IgG pAb was purified by (1) ammonium sulfate precipitation method, (2) caprylic acid purification method, and (3) DEAE anion exchange chromatographic method. After each step, SDS-PAGE was used to analyze the fractions.

#### Ammonium sulfate precipitation method

One hundred grams of pure ammonium sulfate salt was dissolved in 100 ml of dist. H_2_O. After complete dissolving (2 days), the supernatant was separated and the pH was adjusted to 7–7.2 by drops of concentrated ammonia. Saturated ammonium sulfate solution was added dropwise to rabbit anti-*S. mansoni* serum to reach 50% saturation, with continuous stirring followed by centrifugation for 20 min at 4°C. The supernatant was discarded and the ammonium sulfate precipitation was repeated several times on the precipitate.

#### Caprylic acid purification method

Anti-*S. mansoni* IgG pAb, partially purified by ammonium sulfate precipitation, was diluted with 2 volumes of 60 mM Na acetate buffer, pH 4.8. This step may be replaced by dialysis versus 3 mM sodium acetate (pH 4.8). Caprylic acid (7%) was added dropwise with slow magnetic stirring for 30 min at 4°C. The mixture was centrifuged at 1000 g for 30 min. Albumin and other non-IgG proteins were precipitated except IgG. The precipitate was separated while the supernatant (containing nearly pure IgG) was further purified.

#### DEAE Sephadex A-50 ion exchange chromatography

Generally, protein can be separated by DEAE chromatography based on their charge. DEAE group has a +ve charge which can be neutralized by a counter ion, like chloride ions. Since antibodies have more basic isoelectric point than the majority of other serum proteins, they bind weaker to the DEAE group than albumin. Also, IgG is more basic than IgM due to the presence of more lysine and arginine than glutamate and aspartate residues. If the ionic strength was increased by increasing the NaCl salt concentration in the eluting buffer, chloride ions will compete with the bound proteins in the binding to the positive DEAE group and the proteins are eluted. IgG molecules is eluted earlier than IgM and albumin. IgG fractions separated by this method have high degree of purity. One gram of DEAE Sephadex A-50 (Pharmacia, Uppsala, Sweden) powder was swelled in 0.5M Tris buffer (200 ml, pH 7) and washed with 3 bed volumes 20mM Tris buffer, pH 7 five times. The swelled beads suspension was poured in 30×2.5cm column (Bio-Rad) using a glass rod, avoiding air bubbles. Following beads settling in the column, the surface was covered with the binding buffer followed by the determination of the approximate column binding capacity. Samples were dialyzed against the binding buffer (20mM Tris-HCl, pH 7). The outlet tubing of the column was then closed and the buffer above the beads was removed. IgG sample with suitable volume and protein content <10% of column bed capacity was applied to the column using a Pasteur pipette. The outlet tubing was opened until the sample penetrates the beads, and then closed again for 10 min for IgG binding to the beads. The outlet was opened and connected to an automated fraction collector (LKB, Dusseldoef, W Germany); 1 ml fraction was collected in each tube. The absorbance (280 nm) was measured for each fraction using a spectrophotometer (Perkin-Elmer Lambda 1A). Fractions exhibiting high absorbance at first peak were pooled together. The protein content and purity of pAbs were measured by Bio-Rad protein assay and SDS-PAGE, respectively. Testing for reactivity of pAbs was performed by indirect ELISA.

#### pAb labeling with horseradish peroxidase (HRP, periodate method) according to Tijssen and Kurstak (1984)

Five-milligram HRP was dissolved in 1.2 ml dist. H_2_O; then, 0.3 ml sodium periodate was added, and the mixture was incubated at room temperature for 20 min. At 4°C, the HRP mixture was dialyzed many times overnight versus 1mM sodium acetate buffer (pH 4). Five milligrams per milliliter of the pAb IgG was mixed with 0.02M carbonate buffer (pH 9.6). Half milliliter of pAb solution was mixed HRP solution, followed by 2 h of incubation at room temperature. One hundred microliters of sodium borohydride was mixed with the solution, followed by incubation at 4°C for 2 h. The HRP-conjugated pAb was dialyzed versus PBS (0.01M, pH 7.2).

### Application of pAbs

#### Samples’ collection

The study involved 200 patients infected with *S. mansoni* from highly endemic areas and outpatients’ clinic and hospitals, where they were diagnosed by finding characteristic eggs in the collected stool samples, in addition to 100 patients that were infected with other parasites (*Fasciola*, hookworm, hydatid, and trichostrongyloids) and 100 healthy negative control individuals. Samples (serum and stool) were collected and kept at −80°C until used. The study was conducted in accordance with the World Medical Association Declaration of Helsinki for human subjects and all participants gave an informed written consent.

#### Parasitological examinations (stool examination)

Microscopic examination of stool samples was performed by merthiolate-iodine-formaldehyde concentration technique (MIFC) according to Blagg et al. (1955) and egg count using Kato-Katz technique (Engels et al. 1997).

#### Detection of *S. mansoni* Ags by sandwich ELISA in serum and stool samples

##### Standardization of sandwich ELISA

The IgG pAb maximum concentration as a coating and peroxidase conjugated layer was 10 and 1/20 μg/ml, respectively. The assay was performed according to Qiu et al. (2000), Hegazy et al. (2015), and Kamel et al. (2013).

##### Optimization of working dilution of stool samples

One part of sample was mixed with two parts of dist. H_2_O in a 15-ml polypropylene tube, followed by well stirring and centrifugation for 10 min to prepare aqueous elutes from each sample. Serial dilution of diluted stool (the supernatant) was assessed for Ag detection by sandwich ELISA to obtain maximum values with minimal background reaction. Five dilutions (1:10, 1:20, 1:30, 1:40 and 1:50 in 2.5% FCS in PBS/T) of diluted stool (diluted 1:3 before) were used.

##### Sandwich ELISA

Plate was coated with 100μl/well of purified IgG pAb at a concentration of 10 μg/ml followed by incubation at room temperature overnight. Plate was washed three times with washing buffer (0.1 M PBS, 0.05% Tween (v/v), pH 7.2). The plate was blocked by adding 200μl/well of blocking buffer (2.5% FCS in PBS/T); followed by incubation for 2 h at 37°C. After plate was washed three times, samples (100 μl/well) were added to the wells followed by 2 h of incubation 37°C, then washing. One hundred microliters/well of HRP-conjugated pAb IgG (diluted 1/20 for IgG) was added to the plates. The plates were incubated for 1 h at room temperature, followed by five times washing. One hundred microliters of substrate was added to each well, and the plate was incubated for half an hour in darkness at room temperature, followed by the addition of 50 μl/well H_2_SO_4_ to stop the reaction (Farid et al. 2022a). The absorbance was estimated by ELISA reader at 492nm.

Note: Substrate was prepared by mixing OPD, dissolved in 25ml of 0.05M phosphate-citrate buffer (pH 5), with 5 μl H_2_O_2_.

#### Validity of results

Diagnostic sensitivity of a method refers to the frequency of positive test results detected by a particular method in individuals with a particular disease. Therefore, the higher percentage of the test sensitivity, the higher the number of positive results in diseased individuals.

$$\textrm{Sensitivity}\ \left(\%\right)=\left[\textrm{true}+\textrm{ve}\ \textrm{cases}/\left(\textrm{true}+\textrm{ve}\ \textrm{cases}+\textrm{false}\hbox{--} \textrm{ve}\ \textrm{cases}\right)\right]\times 100$$

Diagnostic specificity of a method refers to the frequency of negative test results detected by a particular method in individuals without the particular disease. Therefore, the higher percentage of the test specificity, the higher the number of negative cases in healthy individuals.

$$\textrm{Specificity}\ \left(\%\right)=\left[\textrm{true}-\textrm{ve}\ \textrm{cases}/\left(\textrm{true}-\textrm{ve}\ \textrm{cases}+\textrm{false}+\textrm{ve}\ \textrm{cases}\right)\right]\times 100$$

The mean percentages of positive results that were true positive and negative results that were true negative were estimated by PPV (positive predictive value) and (NPV) negative predictive value, respectively.

$$\textrm{PPV}\ \left(\%\right)=\left[\textrm{true}+\textrm{ve}\ \textrm{cases}/\left(\textrm{true}+\textrm{ve}\ \textrm{cases}+\textrm{false}+\textrm{ve}\ \textrm{cases}\right)\right]\times 100$$

$$\textrm{NPV}\ \left(\%\right)=\left[\textrm{true}-\textrm{ve}\ \textrm{cases}/\left(\textrm{true}-\textrm{ve}\ \textrm{cases}+\textrm{false}-\textrm{ve}\ \textrm{cases}\right)\right]\times 100$$

#### Statistical analysis

The data were presented as mean± standard deviation (SD). Correlation between number of *S. mansoni* eggs in stool sample and the optical density (OD) of ELISA technique was estimated by correlation coefficient (*r*) according to Snedecor and Cochran (1981). The data were considered significant if *P* <0.05.

## Results

### CP purification

#### DEAE Sephadex A-50 ion exchange chromatography

**Fig. 1:**
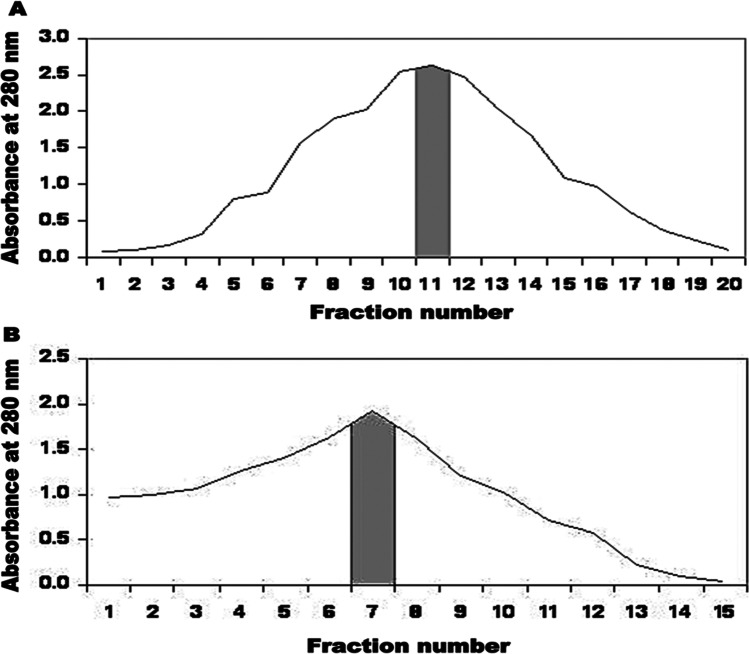
Elute profile for chromatography of E/S products on DEAE Sephadex A-50 ion exchange chromatography (**A**) and Sephacryl S-200 column (**B**)

At fraction number 11, single maximum peak (2.645) represented the antigen (Fig. 1A).

#### Sephacryl S-200 column chromatography

**Fig. 2:**
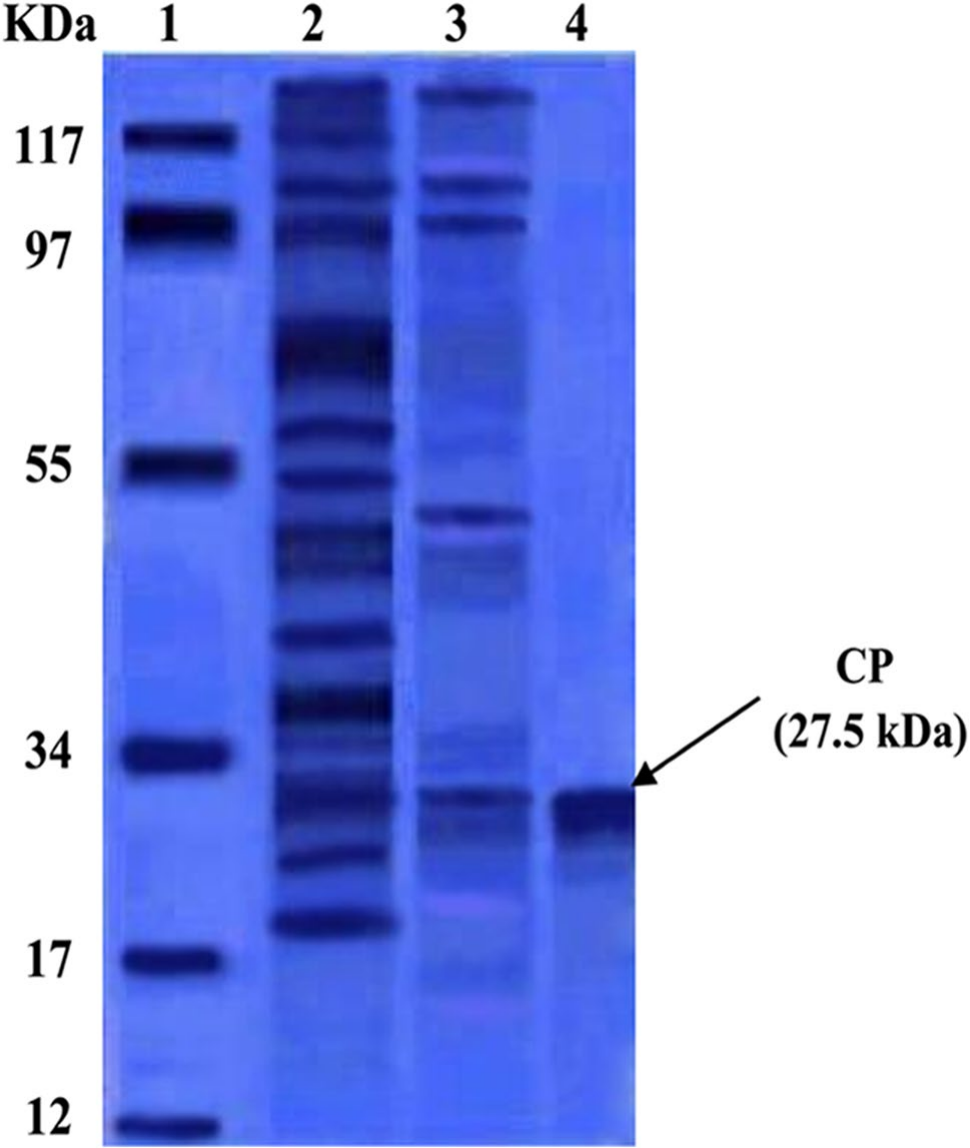
SDS-PAGE of target E/S Ags eluted from affinity chromatography columns (stained with coomassie blue). **Lane 1:** Low molecular weight standard. **Lane 2:** E/S products. **Lane 3:** Target Ag eluted from DEAE Sephadex A-50 ion exchange chromatography. **Lane 4:** Target Ag eluted from Sephacryl S-200 gel filtration chromatography

The resulted E/S antigen fraction number 11 was further processed by Sephacryl S-200 column chromatography. The obtained single peak, at fraction number 7 with OD280 value 1.910, represents the column elution volume fractions which comprised cysteine proteases (Fig. 1B). The fractions of the eluted protein, from the previous purification techniques, were evaluated by 12.5% SDS-PAGE and revealed just single band at 27.5 kDa that represented cysteine proteases (Fig. 2).

#### Measurement of total protein content of *S. mansoni* CP Ags

Total protein was 8 mg/ml in the collected E/S product from the adult worms, and 4.6 and 2.3 mg/ml after purification with DEAE Sephadex A-50 ion exchange and Sephacryl S-200 chromatography, respectively.

#### Characterization of *S. mansoni* Ag

*S. mansoni*-infected patients’ sera samples showed a vigorous response against *S. mansoni* CP antigen with mean OD492 equivalent to 1.309. No cross reactions were detected with patients’ serum infected by *Fasciola*, hydatid, and hookworm (Table 1).

**Table 1:** Reactivity of purified target CP Ag by indirect ELISA

OD readings at 492 nm as mean (SD)	Serum samples
1.309 (0.342)	*S. mansoni*
0.264 (0.201)	*Fasciola*
0.106 (0.094)	Hydatid
0.182 (0.082)	Hook worm

*OD*, optical density; *SD*, standard deviation

#### Production of pAbs

**Fig. 3:**
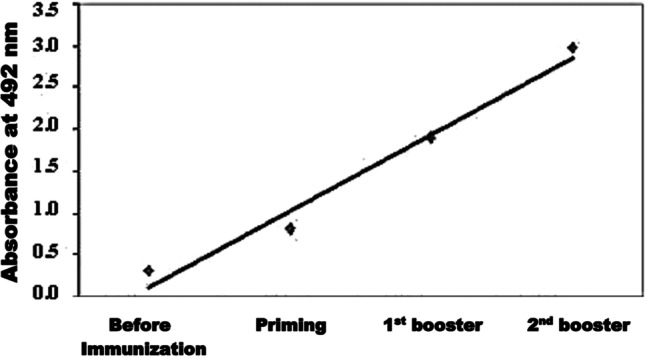
Reactivity of rabbit anti-*S. mansoni* antibodies (diluted 1/250) against *S. mansoni* CP by indirect ELISA

Before the injection of each immunizing dose, blood samples from New Zealand white rabbit were collected. Indirect ELISA was used for testing the presence of specific anti-*S. mansoni* antibodies. An elevating antibody level was recorded started 1 week after the first booster dose. Three days after the second booster dose, immune serum samples gave strong high titers against *S. mansoni* CP with OD492 of 2.8 (1/250 dilution) (Fig. 3).

#### Polyclonal antibodies purification

**Fig. 4:**
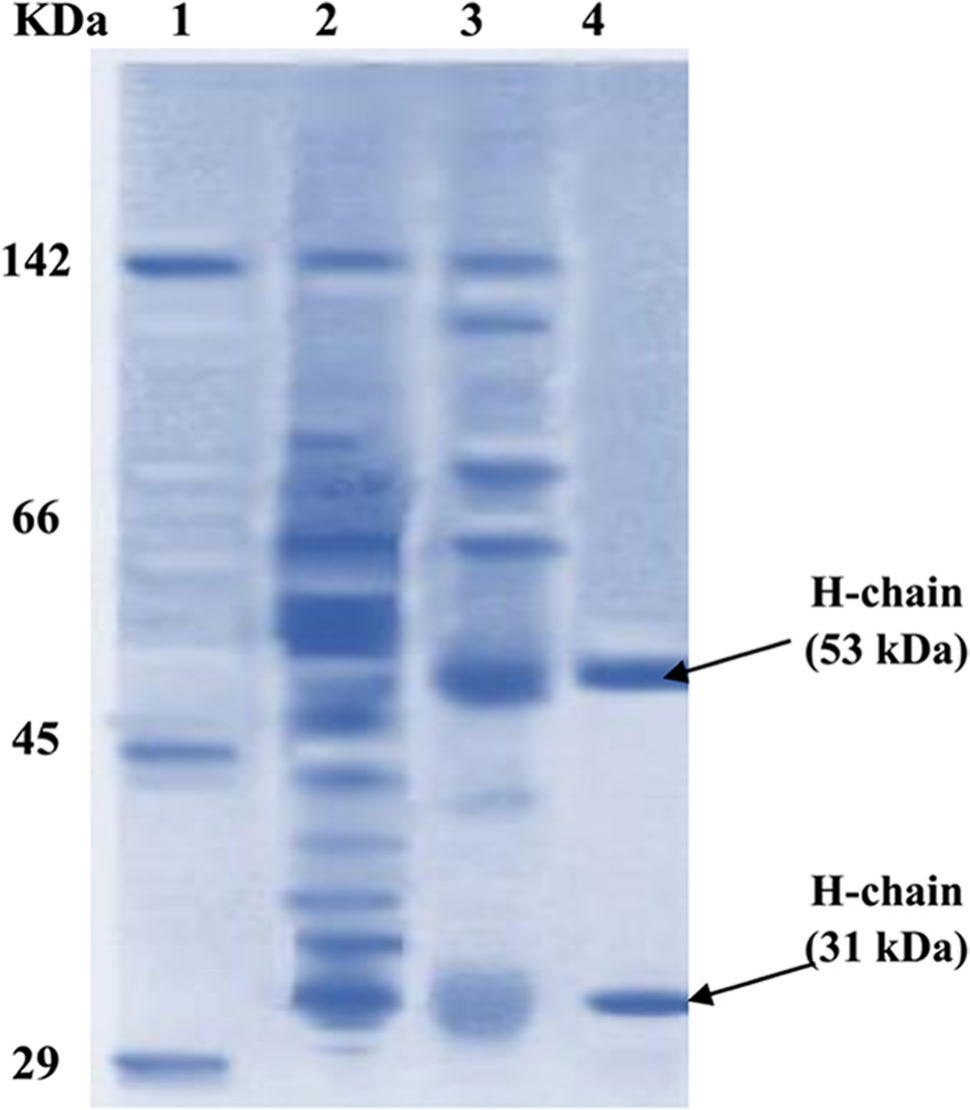
12.5% SDS-PAGE of anti-*S. mansoni* IgG pAb before and after purification (stained with coomassie blue). **Lane 1:** Molecular weight of standard protein. **Lane 2:** Precipitated proteins after 50% ammonium sulfate treatment. **Lane 3:** Nearly pure IgG pAb after 7% caprylic acid treatment. **Lane 4:** Purified IgG pAb after ion exchange chromatography

Crude rabbit’s serum had a total protein content of 12.5 mg /ml. The produced purified anti-*S. mansoni* IgG pAb was measured from the evaluation of the protein content after each purification stage. The protein content was 5.8 mg/ml using the 50% ammonium sulfate precipitation form, while the content decreased to 3.1 mg/ml after a 7% caprylic acid precipitation procedure. Eventually, the extremely pure anti-*S. mansoni* IgG pAb after chromatography was 2.4 mg/ml. The eluted IgG was expressed at fraction number 10 by a single OD peak (2.88). Analysis by 12.5 % SDS-PAGE revealed that precipitated proteins appeared as several lines. The pure IgG pAb was expressed at 53 and 31 kDa that represent the heavy (H) and light (L) chain pairs. The pAb showed up free of other proteins (Fig. 4).

#### Specificity of purified antibodies

The produced anti-*S. mansoni* IgG pAb gave high reactivity to *S. mansoni* CP. The OD492 for *S. mansoni* were 2.91 compared to 0.432, 0.210, 0.426, and 0.271 for other parasite (Table 2).

**Table 2:** Reactivity of rabbit anti-*S. mansoni* antibodies against many parasitic Ags by indirect ELISA (OD reading= 492 nm)

OD readings at 492 nm	Parasitic Ag
2.84 (0.45)	*S. mansoni*
0.462 (0.13)	*Fasciola*
0.310 (0.22)	Hookworm
0.406 (0.34)	Hydatid
0.281 (0.24)	Trichostrongyloids

*OD*, optical density

### Study population

#### Parasitology investigation

According to stool analysis, 185 patients from 200 patients were positive with *S. mansoni* ova. Therefore, the sensitivity of parasitological diagnosis technique was 92.5%. According to the infection intensity, patients were divided into two subgroups: Light infection that included 90 patients with number of ova/three slides of stool ranging from 6 to 25 ova with mean of 16±9.8. Heavy infection that included 95 patients with number of ova/three slides of stool more than 25 ova with mean of 42±17.91.

### Sandwich ELISA

#### Measurement of *S. mansoni* CP Ags in stool samples (coproantigens)

The positivity cutoff value equaled 0.486. As shown in Table 3, the OD492 value of *S. mansoni-*infected patients’ group (1.86±0.317) was higher than that of the uninfected control group (0.290±0.098), and that of the other parasite-infected patients’ groups (Table 3). Three patients out of 200 *S. mansoni-*infected patients gave false negative results. Those patients were from the highly infected subgroup where the ova number was 9±0.201.

**Table 3:** Detection of coproantigens in stool samples of infected human

Group (no of cases)	Positive cases		Negative cases	
	*No.*	*X* **(***SD***)**	*No.*	*X* **(***SD***)**
Healthy control *(n= 100)*	-	-	100	0.290 (0.098)
*S. mansoni (n= 200)*	197	1.86 (0.317)	3*	0.406 (0.070)
*Fasciola (n= 25)*	1**	0.509 (0.069)	24	0.332 (0.109)
*Hookworm (n= 25)*	-	-	25	0.297 (0.185)
*Hydatid (n= 25)*	1**	0.594 (0.121)	24	0.317 (0.185)
*Trichostrongyloid (n= 25)*	-	-	25	0.301 (0.128)

*X*, mean; *SD*, standard deviation. *False negative result. **False positive result

So the sensitivity of the assay was 98.5%. All the OD492 values of 100 uninfected controls were lower than the cutoff value, while the OD492 values of 2 patients from 100 other parasite-infected patients were higher than the cutoff value giving 99.0% specificity. The PPV was 98.9% while the NPV was 98.5% (Table 5).

#### Measurement of *S. mansoni* CP Ags in serum samples

The positivity cutoff value was 0.4. The OD492 of *S. mansoni-*infected group (1.53±0.308) were more than those of the uninfected control group (0.198±0.101) and the other parasite-infected groups (Table 4). Four patients out of 200 *Schistosoma-*infected patients showed false -ve result and the sensitivity was 98.0%. The OD492 of control individuals were lower than the cutoff value while three patients of 100 other parasite-infected patients showed OD492 value higher than the cutoff value giving a specificity of 98.5% (Table 5).

**Table 4:** Detection of circulating *S. mansoni* Ag in serum of human subjects infected with *S. mansoni* or other parasite in comparison to healthy control

Group (no of patients)	Positive cases		Negative cases	
	*No.*	*X* **(***SD***)**	*No.*	*X* **(***SD***)**
*Healthy control (n= 100)*	-	-	100	0.198 (0.101)
*S. mansoni (n= 200)*	196	1.53 (0.308)	4*	0.300 (0.081)
*Fasciola (n= 25)*	2**	0.641 (0.243)	23	0.244 (0.154)
*Hookworm (n=25)*	-	-	25	0.201 (0.112)
*Hydatid (n= 25)*	1**	0.436 (0.209)	24	0.195 (0.200)
*Trichostrongyloid (n= 25)*	-	-	25	0.209 (0.094)

*X*, mean; *SD*, standard deviation. *False negative result. **False positive result

**Table 5:** Sensitivity, specificity, PPV, and NPV of sandwich ELISA used for detection of *S. mansoni* Ags in stool and serum samples

Samples		Sensitivity	Specificity	PPV	NPV
Human	Stool	98.5%	99.0%	98.9%	98.5%
	Serum	98.0%	98.5%	98.4%	98.0%

**Fig. 5:**
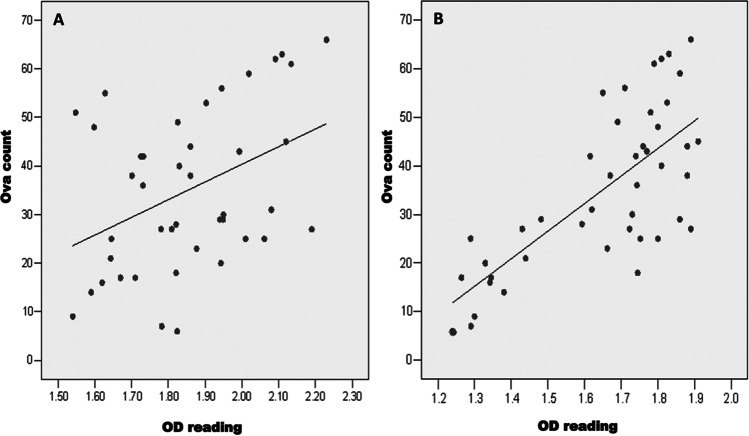
**A** Correlation between ova count/gm stool and *S. mansoni* CP Ag level in stool (coproantigen) of *S. mansoni-*infected patients (*r*= 0.417; P= 0.01**). **B** Correlation between ova count/gm stool and *S. mansoni* Ag level in serum of *S. mansoni-*infected patients (*r*= 0.687; P= 0.001***)

In *S. mansoni*-infected patients, there was a strong positive correlation between the number of ova per gram stool and coproantigen level (Fig. 5A; *r*=0.417 and *P=0.01*), or Ag level in serum (Fig. 5B; *r*=0.687 and *P=0.001*).

## Discussion

Schistosomiasis is a major parasite illness in many regions of the tropics, second only to malaria (van der Werf et al. 2003; McManus et al. 2018). Agricultural practices transmit schistosomiasis, especially when there is inadequate sanitation and continuous contact with water (McManus and Loukas 2008). *Schistosoma* spp. continually excrete and secrete chemicals (which are known as E/S) into their surroundings during skin penetration, either to facilitate passage or as part of their metabolism. Different kinds of proteins and lipids have been found in E/S products (Liu et al. 2009). Proteomic study of the tegument of adult worms, E/S products, and egg shells of *S. mansoni* produced several thousand proteins, according to Braschi et al. (2006) and Liu et al. (2006). Proteases are one of the main components of excretory/secretory products, and they have a significant importance in all living forms (Barrett et al. 2004; McKerrow et al. 2006).

By culturing adult worms in vitro, E/S product has been collected to be analyzed, where proteases were found to constitute a high percent. Proteases play an important role in all living organisms (Barrett et al. 2004). Hatching, excystment, tissue-cell invasion, nutrition acquisition, and immune evasion are all processes that require the use of proteases (McKerrow et al. 2006). Proteases function at the host/parasite interface for trematode worms that cause diseases of veterinary and medical consequence, aiding movement, digesting protein molecules, and likely immune evasion (Dalton et al. 2006). Many parasites rely on CPs for their metabolism, and CP inhibitors have been proven to eliminate protozoan parasites in both culture (Rosenthal, 2004; Verma et al. 2016) and animal disease models (Rosenthal, 2004; Farid et al. 2013).

Schistosomes have a variety of CPs that help in nutrition, reproduction, and proteins’ turnover (Caffrey et al. 2000). CP inhibitors (fluoromethyl ketone) have been demonstrated to reduce worm and egg loads in *S. mansoni*-infected mice (Caffrey et al. 2004).

In the present study, adult *S. mansoni* worms were cultured in RPMI 1640 medium, and E/S products had been collected in order to purify and analyze CPs. CPs were purified from excretory/secretory products by ion exchange and gel filtration chromatography and in which CP appeared as one band at 27.5 kDa by SDS-PAGE. Excretory/secretory products generated by worms have long been thought to be a significant source of antigens capable of eliciting an immunological response in their hosts (Wasilewski et al. 1996). Some excretory/secretory products have been linked to specific secretory and excretory organs. Molecules generated from the helminths tegument, on the other hand, contribute significantly to the contents of the parasite’s E/S products in vitro, and are likely also discharged in vivo (Bobardt et al. 2020). In vitro and in vivo, surface components of *S. mansoni* schistosomules and mature worms were also shown to be shed (Ryan et al. 2020). Through their possible triggering effect on the immune response of susceptible hosts, the active release of antigens from the surface of helminths should have a potentially major impact on the experimental therapeutics of the helminth/host connection (Cutts and Wilson 1997).

In this study, anti*-S. mansoni* IgG pAb was prepared by immunization of rabbit with *S. mansoni* E/S Ags. Each rabbit recieved 1 mg of *S. mansoni* proteinase worm extract in the first dose and half milligram for the 2^nd^ and 3^rd^ booster dose injection. Two weeks following the priming dose, the first boosting dose was given, where according to Tendler et al. (1991), the appropriate boosting doses were administered at weekly intervals. The purification processes used in this work were acceptable; 3 purification stages were used for IgG pAb: ammonium sulfate precipitation, 7% caprylic acid precipitation, and lastly ion exchange chromatography. The quality of IgG pAb was determined using a 12% SDS-PAGE; the heavy and light chain bands of pure pAb IgG were 53 and 31 kDa, respectively, indicating that the purified Ab seems to be devoid of unrelated proteins. By these procedures, the output of pAb as protein content was 2.3 mg/ml from an initial protein content of 12.5 mg/ml in the excretory/secretory products. Compared to the amount of pure immunoglobulin obtained from any biological sample using similar separation techniques, the yield was reasonable (Ayón-Núñez et al. 2018; Bride et al. 1995). Then, using an indirect ELISA against E/S products, the response of the pure pAb towards *S. mansoni* CP and other parasites’ Ag (*Fasciola*, hookworm, hydatid, and trichostrongyloids) was assessed using the Engvall and Perlmann (1971) method with some modifications of Voller et al. (1974). It is worth noting that ELISA has been regarded as a reliable assay for detecting antibodies to fluke antigens in rabbits. As an immunodiagnostic approach for numerous parasite illnesses and for quantitative evaluation of many immunological markers, the ELISA has received the greatest attention in many studies (Abdel-Monaem et al. 2015; Madbouly et al. 2021; Farid et al.*,* 2022b).

In this study, the produced anti-*S mansoni* IgG pAb was utilized for the detection of CP Ags in stool (coproantigens) and sera samples of infected patients by sandwich ELISA. The standardization of different reagents used in sandwich ELISA was undertaken by testing different concentrations of one reagent in the presence of fixed excess amount of the other reagents. The highest concentration of pure IgG PAb as a coating layer was 10 μg/ml, whereas the highest dilution of IgG PAb as a peroxidase-conjugated layer was 1/20 μg/ml. Moreover, the standard curve was constructed for IgG pAb against different concentrations of E/S CP Ag. The curve was linear based on the concentration of Ag. The lowest detecting concentration for E/S was 2.3 ng/ml.

Also, the study compared the ordinary parasitological examination methods with sandwich ELISA in the diagnosis of schistosomiasis. The study was conducted on 200 *S. mansoni-*infected patients, 100 patients infected by other parasites, and 100 healthy volunteers. According to parasitological examination and the egg load in stool (counted by Kato technique), 185 patients from 200 patients were positive with *S. mansoni* ova. Samples were distributed into two main groups: the first group represented light infection (< 40 ova/g stool) where the mean egg counts were 16 ± 9.8 in infected human; the second group represented heavy infection (> 40 ova/g stool) and the mean egg counts were 42 ± 17.91 ova/gm stool for infected human. The sensitivity of that test was 92.5%.

The rabbit IgG raised against purified 27.5 kDa *S. mansoni* CP Ag successfully detected CP Ags in stool samples of patients with a sensitivity of 98.5% and specificity of 99.0%, respectively. Although all of the negative control stool samples showed a negative reaction, one patient with *Fasciola* and another patient with hydatid infection showed cross reactivity. Three patients infected with *S. mansoni* had false negative results. The test had a 98.9% PPV and a 98.5% NPV, respectively. To detect circulating *S. mansoni* antigens, serum samples were also used in a sandwich ELISA experiment. Although none of the negative control sera samples showed a positive response, two patients with *Fasciola* and another patient with hydatid infection showed cross reactivity. The test specificity was 98.5%. Four cases of *S. mansoni*-infected patients, on the other hand, yielded a false negative result; the test’s sensitivity was 98.0%. The test has a PPV of 98.4% and a NPV of 98.0%, respectively.

Both stool and serum antigen concentrations were found to have a positive connection with *S. mansoni* egg load. Because the density of flukes in the host affects the stool egg count, it is reasonable to assume that stool antigen levels in *S. mansoni*-infected patients were directly proportional to the number of adult worms. It is possible that the absence of parasite antigen in certain patients’ stools and sera was due to a low parasite burden, resulting in undetectable concentrations of antigen in the stool and sera. A similar link has also been observed in *S. mansoni*-infected patients in several studies (Grenfell et al. 2013).

Controlling schistosomiasis requires accurate and timely diagnosis, which includes early diagnosis and subsequent population therapy, measurement of morbidity, and assessment of control programs (Peng et al. 2008). Thus, it can be concluded that purified 27.5 kDa CP obtained from *S. mansoni* E/S products can be used as an appropriate applicant antigen for immunodiagnosis. Moreover, the purified IgG pAb successfully detects CP Ags in stool and sera samples of *S. mansoni-*infected patients with light infection. This study reported that, sandwich ELISA has a higher sensitivity (98.5% in stool samples and 98.0% in serum samples) than microscopic examination of eggs in stool (92.5%).

Based on our findings, we infer that CP antigen detection can either substitute parasitological testing or be utilized as a supplemental screening tool in the situation of weak infection. Moreover, CP antigen determination is the preferred method for determining treatment and diagnosing active infection in endemic areas. Moreover, identification of a *S. mansoni* Ag that elicits an intense humoral response in human cases is important for the goal of obtaining better diagnostic tool. E/S Ag as shown in the present study was a sensitive and specific Ag in the different immunodiagnostic techniques used even in its crude form which could be prepared by an easy and simple method and in adequate amount.

## Data Availability

All data generated or analyzed during this study are included in this published article.
